# Experimental Study on a Microwave Composite Forming Process Based on a SiC Mold for Manufacturing Fiber Metal Laminate

**DOI:** 10.3390/ma14195547

**Published:** 2021-09-24

**Authors:** Eu-Tteum Park, Jeong Kim, Beom-Soo Kang, Woojin Song

**Affiliations:** 1 Department of Aerospace Engineering, Pusan National University, Busan 46241, Korea; bangsbang91@pusan.ac.kr (E.-T.P.); greatkj@pusan.ac.kr (J.K.); bskang@pusan.ac.kr (B.-S.K.); 2 Department of Nanomechatronics Engineering, Pusan National University, Busan 46241, Korea

**Keywords:** fiber metal laminate, self-reinforced polypropylene, uniaxial tensile test, silicon carbide mold, microwave composite forming

## Abstract

The microwave composite forming (MCF) process can reduce manufacturing cost because the process time is reduced by the dielectric heating of the mold and the composite material. In a previous study, the MCF process using a commercial microwave oven with a polytetrafluoroethylene mold was applied. Disadvantages of the previous MCF process have been investigated. These included the difference in tensile properties according to the cutting location, absence of a method to measure temperature during the MCF process, and the fact that the input power cannot be controlled according to the temperature. To solve these problems, a microwave oven with a silicon carbide mold was proposed in this study. Uniaxial tensile tests were conducted to obtain the tensile properties of the fiber metal laminate (FML) specimen. In addition, a microscopic image was captured to investigate the non-adhesive area. The tensile properties and thickness distribution of the FML specimens manufactured by the proposed and previous MCF processes were compared according to the cutting location of the FML sheets. Furthermore, the non-adhesive area was quantified to compare the processes. The results revealed that the proposed MCF process improved the tensile properties of the FML specimen and reduced the non-adhesive area.

## 1. Introduction

Composite materials have been used widely in various industries such as construction, automotive, and aerospace [1,2]. In particular, fiber metal laminates (FMLs, composed of aluminum sheets on the outside and fiber-reinforced composites on the inside) have been studied because these offer improved chemical resistance, flame resistance, specific strength, etc. [3,4]. Recently, it has become important to manufacture eco-friendly products following stringent environmental regulations. FMLs based on thermoplastic resins have been used because these exhibit remarkable recyclability, specific strength, chemical resistance, and impact absorption performance [5,6,7]. Because of these advantages, FMLs are currently used in the structure of aircraft [8]. Furthermore, research to replace the impact beams of automobiles with FMLs is progressing [9]. However, their high production cost is an issue, given that the overall composite material market (including that of FMLs) is capital-intensive. Therefore, it is important to use cost-effective raw materials to manufacture FMLs and reduce their process time. Considering this, in this study, a microwave composite forming (MCF) process was proposed to reduce the process time.

The MCF process is a composite forming process wherein the composite material and mold are heated by microwave irradiation inside a chamber. The microwave heating principle is based on dipolar heating loss, conduction heating loss, hysteresis heating loss, and eddy current heating loss [10]. Dipolar heating loss is a type of heating loss that occurs when a periodically varying electromagnetic field used for microwave irradiation induces the vibration of dipoles, and thereby, generates frictional heat [10]. Conduction heating loss is a type of heating loss of metallic materials. Free electrons move owing to a periodically varying electromagnetic field during microwave irradiation, and their repetitive movement generates frictional heat [10]. Hysteresis heating loss is one type of heating loss of magnetic materials. When a material is subjected to microwave irradiation, the original magnetic field of the material varies. After the microwave irradiation is terminated, the magnetic field irreversibly returns to its initial state, and a part of the magnetic energy is converted to heat energy [10]. Eddy current heating loss can be generated on the surface of a material if its thickness is larger than the penetration depth of the microwaves. The direction of the eddy current depends on the periodically varying magnetic field during microwave irradiation, and a part of the magnetic energy is converted to heat energy [10]. In accordance with this heating mechanism, the dipolar heating loss can be effective mainly depending on a material of resin, and conduction heating loss can be effective depending on the electrical conductivity of fiber. In addition, when a dielectric material is used in a mold, heat is mainly generated by the conduction heating loss of the dielectric material. If an appropriate heat transfer medium is inserted between the composite material and the dielectric material, heat generated in the mold can be indirectly transferred to the composite material.

Joshi et al. reported that the application of the MCF process based on this microwave heating principle for carbon-fiber-based prepreg reduced the process time and energy consumption by approximately 60% and 25%, respectively [11]. Li et al. compared the mechanical properties of carbon-fiber-reinforced bismaleimide composite manufactured by the autoclave process and MCF process. They reported that the MCF process could fabricate the composite with mechanical properties comparable to those obtained with the autoclave process and reduced the process time and energy consumption by approximately 45% and 3%, respectively [12]. Xu, et al. reported that the MCF process can reduce the processing time of carbon fiber composites by 39% while increasing the compressive strength by 22%, compared with the conventional curing process [13]. Singh et al. studied the effect of microwave power on mechanical properties of coir fiber based on high-density polyethylene composites. They reported that the microwave-optimized process is faster and can obtain effective mechanical properties compared with the conventional heating oven [14]. Kim et al. studied the effect of variations on the thickness of carbon-fiber-reinforced composites during microwave curing. They reported that a temperature overshoot could affect the curing quality of the composites [15]. However, studies on the manufacture of FMLs consisting of self-reinforced polypropylene (SRPP) and aluminum alloys are few.

In a previous study [16], we manufactured a FML consisting of two 5052-H32 aluminum sheets and self-reinforced polypropylene using a commercial microwave oven with a polytetrafluoroethylene (PTFE) mold. However, the commercial microwave oven could not control the temperature of the FML sheet. Furthermore, the PTFE mold applied a non-uniform pressure on the FML sheet because the PTFE plate exhibited non-uniform thermal expansion. The MCF process has another problem, namely, the non-uniform heating of the composite sheet because of the non-uniform electromagnetic field inside the chamber. These problems manifested as differences in the tensile properties with respect to the cutting location of the FML sheet. To solve these problems, a microwave oven with temperature control and uniform pressurization should be manufactured, and a new mold should be considered.

In this study, a microwave oven was manufactured to solve these problems of the commercial microwave oven. In addition, a SiC mold was fabricated to heat the FML sheet uniformly. The SiC mold consists of the SiC plate, heat-resistant blocks, and stainless steel. SiC is a dielectric heating material and has been used in industrial hot air blowers. SiC is heated by dielectric heating loss when irradiated with microwaves. Because the heated SiC indirectly heats the stainless steel of the SiC mold by heat transfer, the FML sheet can be heated relatively uniformly. Therefore, our objective was to manufacture the microwave oven and SiC mold and to compare the tensile properties of FMLs fabricated by this process with those of FMLs fabricated using the commercial microwave oven and PTFE mold proposed in the previous study. First, a preliminary test of the SiC mold was conducted to measure the temperature of the mold and check the generation of spark. Second, uniaxial tensile tests were carried out, and the tensile properties such as tensile modulus, yield strength, and ultimate strength were determined according to the cutting locations of the FML specimens. Third, a plane-section of the FML was photographed using an optical microscope, and the non-adhesive region was investigated. The non-adhesive region of the FML specimen was quantified by calculating the ratio of non-adhesive region (RONAR). Finally, the tensile property RONARs of the FML specimens were compared with those of the FML specimens fabricated with the commercial microwave oven and PTFE mold.

## 2. Materials and Methods 

### 2.1. Specification of Proposed Microwave Oven

#### 2.1.1. Chamber

**Figure 1:**
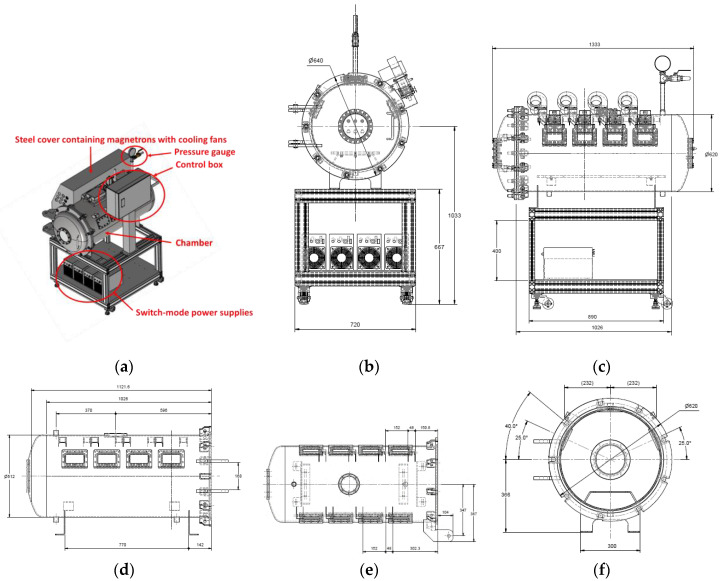
Proposed microwave oven: (**a**) 3D model of microwave oven; (**b**) front view of microwave oven; (**c**) side view of microwave oven; (**d**) side view of chamber; (**e**) top view of chamber; (**f**) front view of chamber.

In this study, a cylindrical chamber with an outer diameter of approximately 620 mm and length of approximately 1026 mm was manufactured, as shown in Figure 1. The chamber was made of stainless steel (STS 304). A dome-shaped door was used at one entrance of the chamber, and the other entrance was sealed by installing a pipe connector for pressurization. The thickness of the chamber was designed to be approximately 6 mm to withstand a maximum internal pressure of up to 15 bar. In addition, two rectangular holes were machined to illuminate the chamber’s interior, and a circular hole was machined so that thermal imaging of the chamber’s interior could be performed. A germanium window was installed at the circular hole to capture infrared images while preventing the leakage of pressurized air and microwaves from the chamber’s interior to the outside. The germanium window can transmit only infrared waves and prevents the leakage of microwaves. Silicate windows were installed in the rectangular holes to illuminate the interior. In addition, a silicate window was installed in the door to visually examine the interior. A steel mesh was installed on the silicate window inside the chamber to prevent microwave leakage from the window. The diameter of the steel mesh was designed to be approximately 2 mm. This value is commonly used in commercial microwave ovens because microwaves cannot penetrate a steel mesh whose diameter is significantly smaller than their wavelength (approximately 122 mm).

#### 2.1.2. Microwave Radiation System

**Figure 2:**
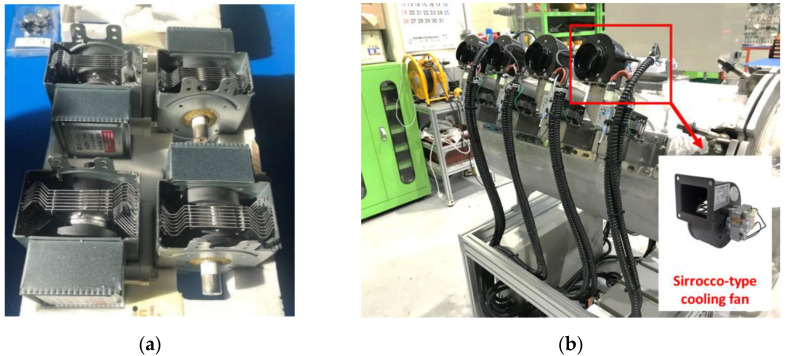
(**a**) Inverter-type magnetrons (2M286-21 GTL); (**b**) Sirocco-type cooling fans installed on each magnetron.

**Figure 3:**
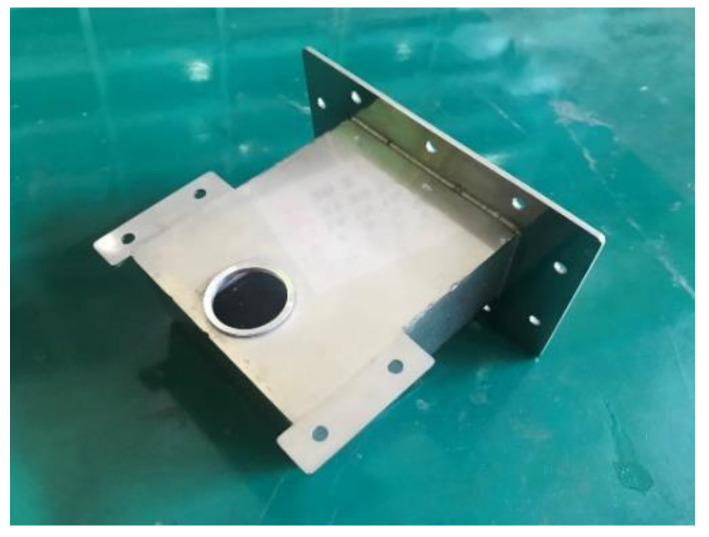
Waveguide (WR 340).

**Figure 4:**
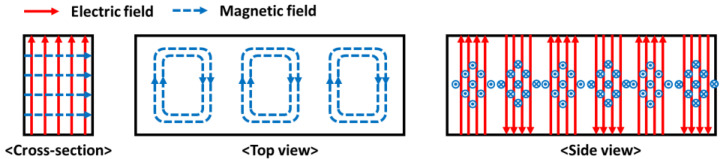
Propagation of TE10-mode microwaves by waveguide.

In this study, four inverter-type magnetrons (2M286-21GTL, Luxtem Co., Seoul, Republic of Korea) that can control input power were used (see Figure 2a). The frequency of each magnetron is 2.45 GHz, and their available input power varies from 0 W to 1100 W. Each magnetron was connected to a switched-mode power supply, which altered the characteristics of the current or voltage to control the input power of each magnetron. A sirocco-type cooling fan was installed to cool each magnetron, as shown in Figure 2b. The waveguide WR 340 was used to transmit the generated microwaves into the chamber, as shown in Figure 3. WR 340 is made of SUS 304 and permits the transmission of transverse electric mode (TE10 mode) microwaves into the chamber. Figure 4 shows the propagation of the TE10-mode microwaves. It is evident that the electric field is perpendicular to the direction of propagation. The waveguide has a cut-off frequency band that does not propagate the microwave depending on the shape of the waveguide. In general, the cut-off frequency band of WR 340 is over 3.471 GHz and below 1.736 GHz [17]. Considering the frequency of the magnetron, WR 340 is suitable for transmitting microwaves into the chamber.

**Figure 5:**
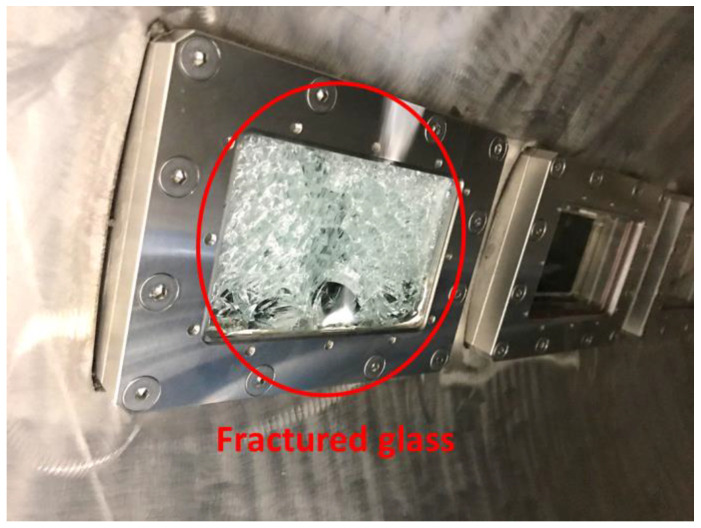
Silicate window fractured owing to dielectric breakdown.

**Figure 6:**
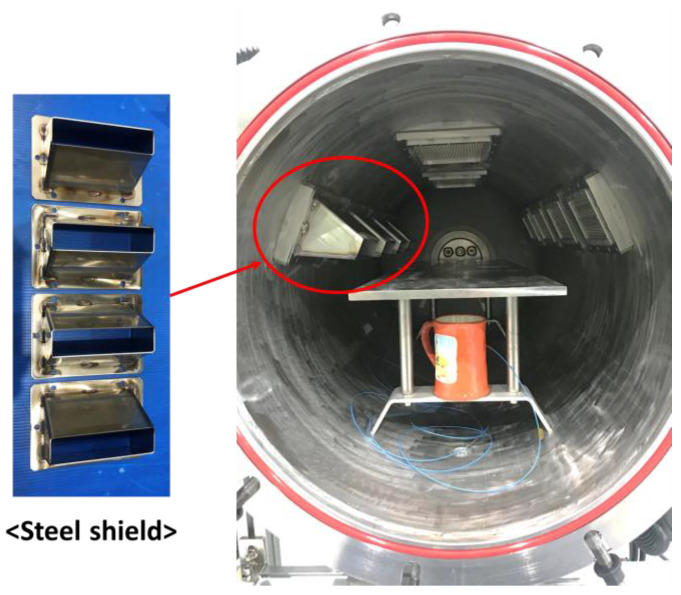
Installation of steel shields to protect each silicate window.

In addition, a silicate pressure window was installed between each waveguide and the chamber to seal the chamber while permitting the transmission of the microwaves. Occasionally, microwaves return to the silicate window, which results in dielectric breakdown of the silicate window (see Figure 5). In this study, four steel shields were installed to prevent dielectric breakdown of the silicate window (see Figure 6). The steel shield was made of SUS 304 and verified to be capable of preventing dielectric breakdown of the silicate window.

#### 2.1.3. Temperature Measurement System

**Figure 7:**
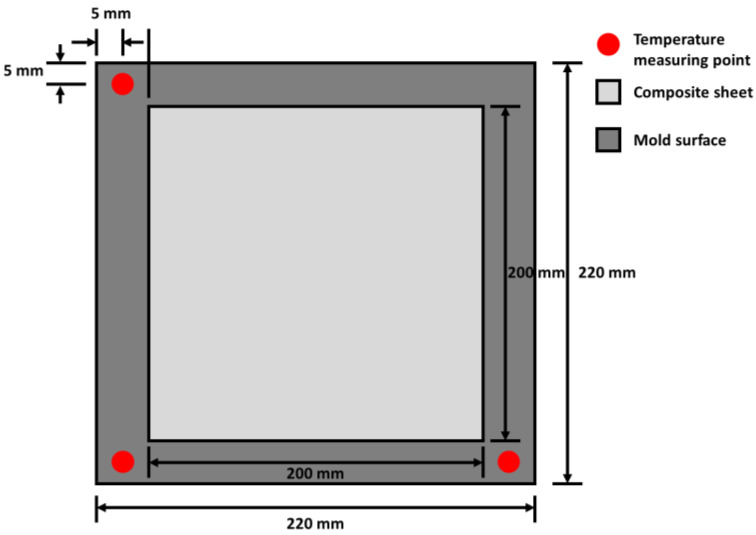
Measurement points of optical fiber temperature measuring sensors.

**Figure 8:**
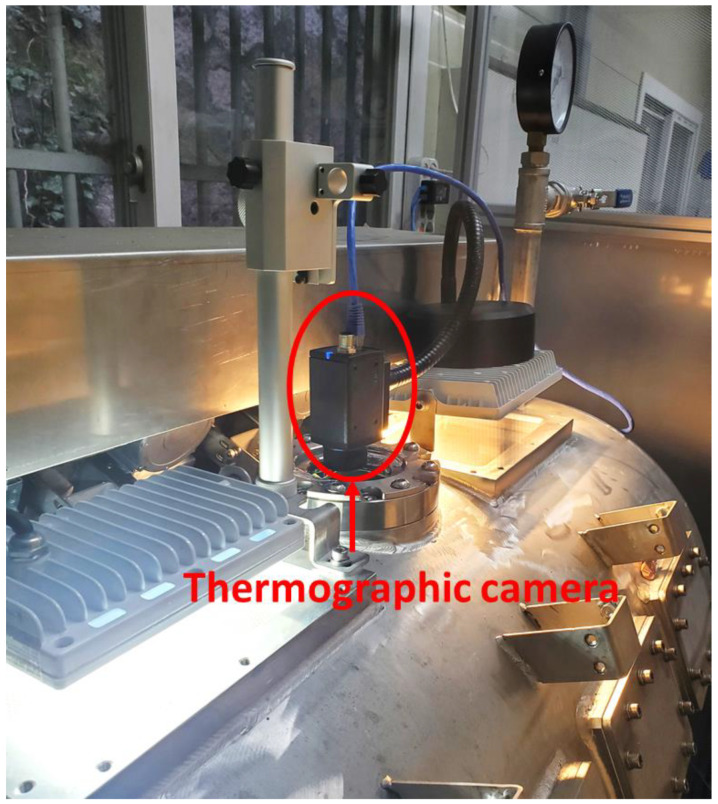
Thermographic camera adjacent to germanium window.

It is necessary to install a temperature measurement system to control the input power and temperature of the mold. When a thermocouple is used to measure the temperature, a spark can be generated at the end of the thermocouple by dielectric breakdown. Therefore, a thermometer with three optical fiber temperature measuring sensors (FOTEMP4-OEM, Weldmann Co., Dresden, Saxony, Germany) was used to measure the temperature of the FML at three points on the mold. Optical fiber temperature measuring sensors can prevent dielectric breakdown by microwaves because their outer shells are made of PTFE. Moreover, these can measure the temperature in a space where an electromagnetic field is generated. The sensors were arranged as shown in Figure 7, and the temperature data captured over time were transmitted to the computer. Furthermore, a thermographic camera (A35SC, FLIR, Wilsonville, OR, USA) was installed to measure the temperature distribution of the FML. Figure 8 shows a thermographic camera mounted on a support.

#### 2.1.4. Temperature Control Method

**Figure 9:**
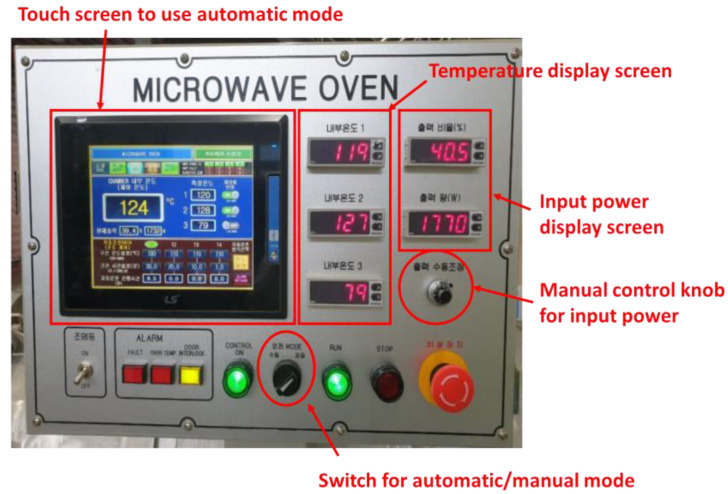
Control board for controlling input power and temperature according to process time.

A control board that can control the input power according to the temperature and process time (see Figure 9) was manufactured and applied to control the temperature of the mold. The input power of each magnetron was set to 1100 W when the temperature was lesser by 15 °C than the set temperature. When the temperature increased, the input power decreased linearly from 1100 to 0 W. When the temperature exceeded the set temperature, the magnetrons stopped working. Furthermore, when the temperature was lower than the set temperature, the input power increased gradually. The control equation for the input power is defined in Table 1.

### 2.2. Manufacture of SiC Mold and Preliminary Test

**Figure 10:**
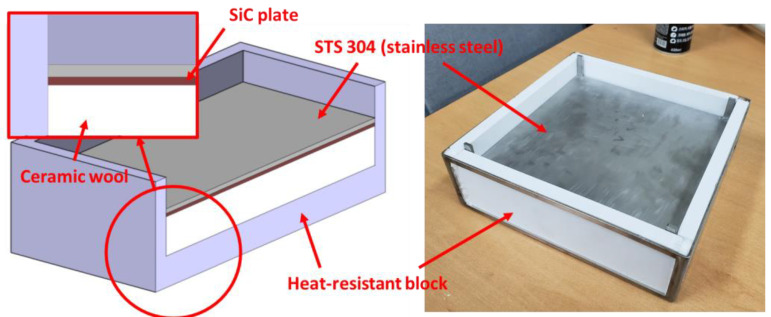
Configuration of the SiC mold.

**Figure 11:**
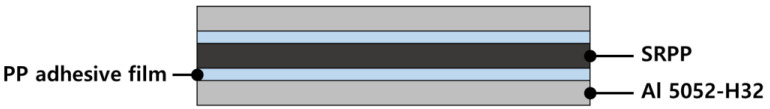
Configuration of the fiber metal laminate.

The SiC mold is composed of heat-resistant blocks, ceramic wool, a SiC plate, and an STS 304 plate, as shown in Figure 10. The SiC plate is a dielectric heating material that is commonly used for heating dental instruments, microwave incinerators, and industrial hot air blowers. Heat-resistant blocks were used to maintain the shape of the mold and protect the chamber from excessive heat. Ceramic wool was used to prevent heat transfer from the SiC plate to the bottom surface of the mold and to mitigate the impact of the SiC plate. The STS 304 plate was used to indirectly transfer heat from the SiC plate to the composite material. The available width and length of the SiC mold are 210 mm, respectively. In this study, the FML consisted of 5052-H32 aluminum, SRPP (Propex Co., Gronau, Germany), and a polypropylene (PP) adhesive film called Collano 23.111 manufactured by Collano AG (Neuenkirch, Luzern, Switzerland, see Figure 11).

**Figure 12:**
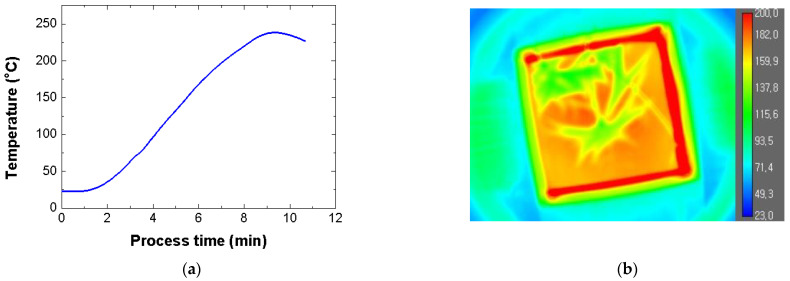
Temperature history and distribution of mold. (**a**) Temperature history; (**b**) Temperature distribution.

The thicknesses of the 5052-H32 aluminum sheet and SRPP sheet were approximately 0.5 mm and 1.0 mm, respectively. The FML specimen for the preliminary test had length and width of approximately 200 mm and 200 mm, respectively. To check the spark and measure the temperature of the FML, the MCF process was conducted for 7 min with an input power of 1100 W. No sparks were generated on the mold or FML specimen. In addition, the temperature of the mold was measured using an optical fiber temperature measuring sensor. Figure 12a shows the temperature history according to the process time of the SiC mold. The SiC mold was heated to approximately 238 °C because of the thermal inertia. Figure 12b shows the temperature distributions of the FML and SiC molds. The temperature of the SiC mold reached 175 °C when the process time was approximately 6 min. This temperature is the melting point of the PP adhesive film [16].

### 2.3. Experimental Procedures

#### 2.3.1. Uniaxial Tensile Test

**Figure 13:**
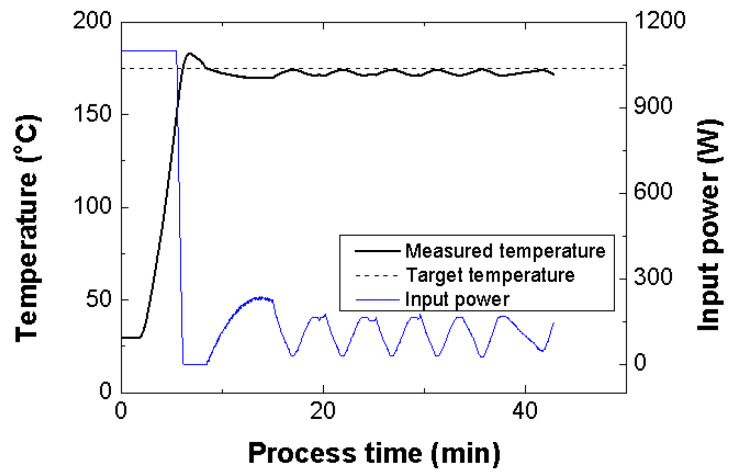
Temperature and input power histories according to process time.

The FML specimens for the uniaxial tensile tests were manufactured using the automatic temperature control method, as shown in Table 1. The set temperature was 175 °C, which is the melting point of the PP film. Moreover, the total process time was 40 min. Before running the MCF process, the space inside the chamber of the proposed microwave oven was pressurized by an air pressure of approximately 0.4 MPa. The input power of the proposed microwave oven was controlled by the average of the temperatures measured by the three optical fiber temperature measuring sensors. The locations of the sensors are shown in Figure 7. Figure 13 shows the average values of the temperature and input power according to the process time. It can be observed that the temperature of the SiC mold increased to approximately 182 °C owing to the thermal inertia of the STS 304 plate. Furthermore, the temperature curve reveals that cooling and heating of the SiC mold occurred repeatedly at approximately 173 °C. Although this temperature is marginally lower than the melting point of the PP adhesive film, the FML can be manufactured effectively with the SiC mold because the process temperature recommended by the manufacturer is 170 °C [18].

**Figure 14:**
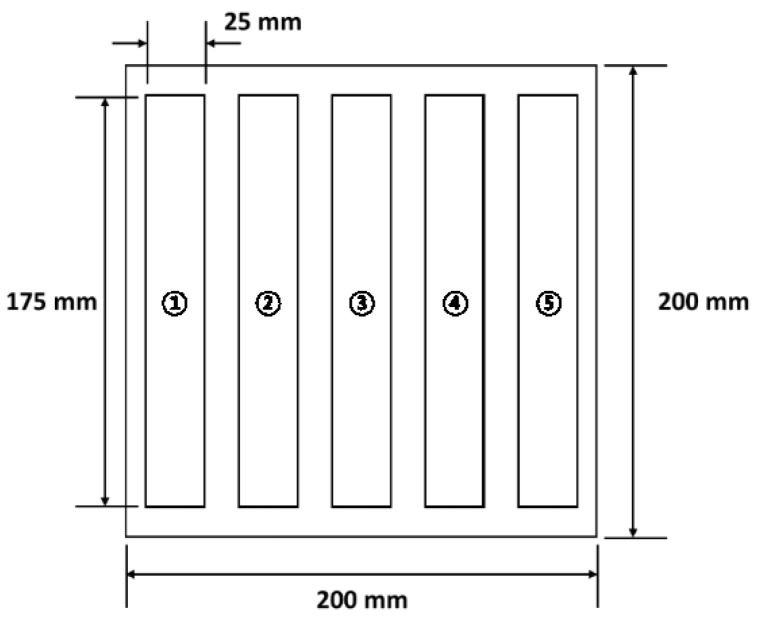
Cutting location on FML specimen.

The FML specimen was manufactured by cutting the FML sheet, as shown in Figure 14. The FML sheet was manufactured with a width and length of approximately 200 mm each considering the size of the SiC mold. The specimens were cut in the longitudinal direction (as shown in Figure 14) to compare the tensile properties of the specimens according to the cutting location on the FML sheet. In the lateral case, 5052-H32 aluminum and woven-type SRPP were used as the constituent materials of the FML sheet. Therefore, the tensile properties that are comparatively identical to the transverse properties were not considered in this study.

The size of the FML specimen was determined according to ASTM D3039 [19]. The specimen was rectangular with a length and width of approximately 175 mm and 25 mm, respectively. A uniaxial tensile test was conducted using a universal material testing machine (Instron 8516). The displacement rate of the FML specimen was 2 mm/min. From the uniaxial tensile test, the load data and displacement data of the FML specimen were obtained, and the stress–strain curve was derived to calculate the tensile modulus, yield strength, and ultimate strength. The tensile properties obtained in this study were compared with those of the FML specimen that was manufactured in the commercial microwave oven using the PTFE mold proposed in the previous study [16].

#### 2.3.2. Microscopic Examination of the Specimens

**Figure 15:**
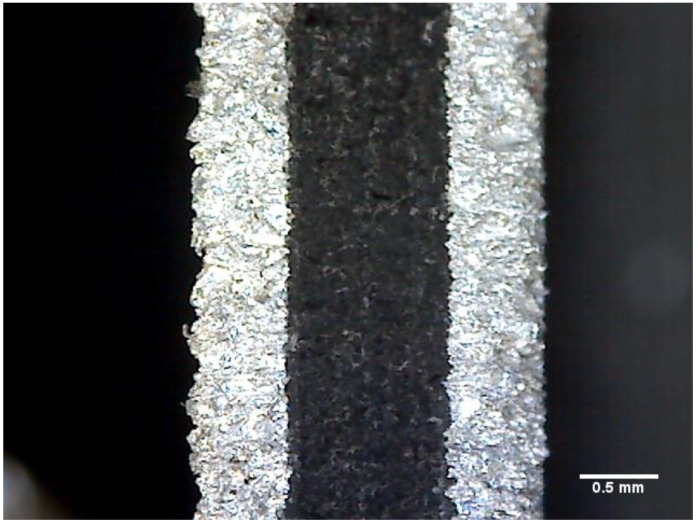
Microscopic image of cross-section of FML specimen at 200× magnification.

**Figure 16:**
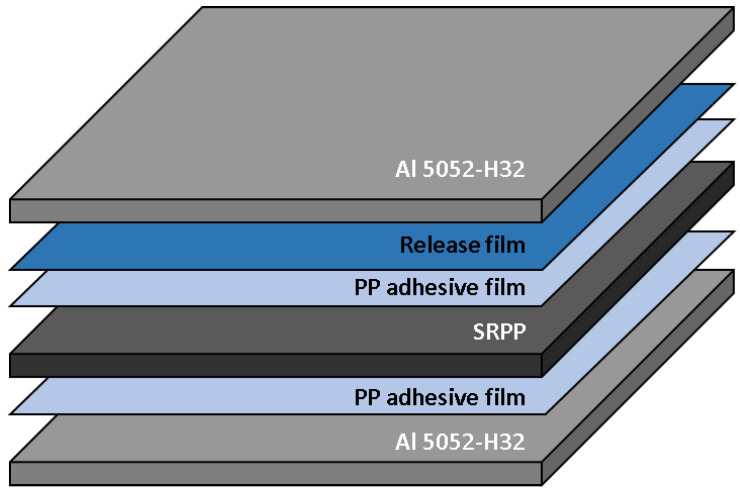
Cutting location of the FML specimen.

**Figure 17:**
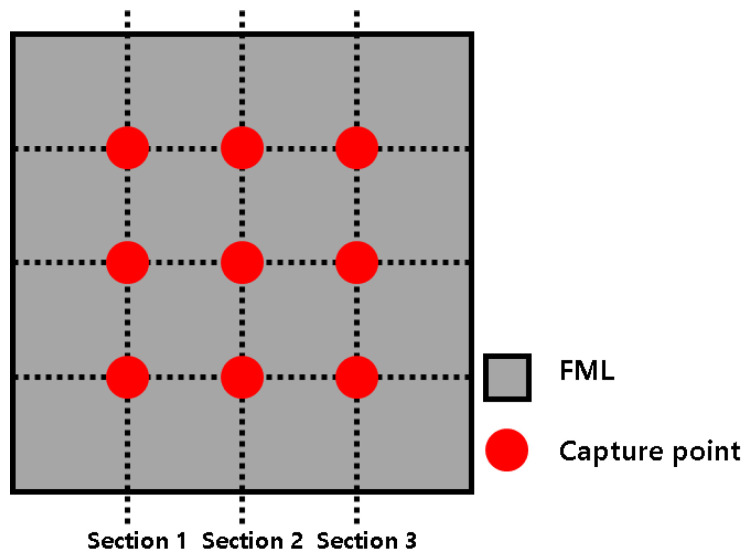
Capture points of the FML specimen.

**Figure 18:**
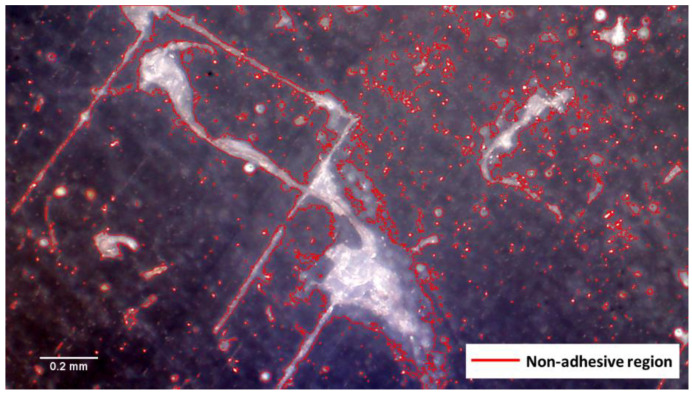
Microscopic image of upper surface of FML specimen and the identified boundary line (400× magnification).

In this study, an optical microscope was used to investigate the non-adhesive area of the FML specimen fabricated using the proposed MCF process. First, the cross-section of the FML specimen was photographed using an optical microscope at 200× magnification (see Figure 15). In the case of the FML specimen, when the PP adhesive film melted, it was difficult to visually distinguish the adhesive layer because it had a transparent color. Therefore, the FML specimen was fabricated by inserting a release film between the aluminum sheet and PP adhesive film, as shown in Figure 16. After fabricating the FML specimen, the upper aluminum sheet and release film were removed to photograph the upper surface of the FML specimen. A microscopic examination of the upper surface was performed using an optical microscope at 400× magnification at nine locations, as shown in Figure 17. An image filtering technique was applied to create a boundary line for the non-adhesive area in the image captured with the optical microscope (see Figure 18).

To quantify the non-adhesive region of the FML specimen manufactured by the MCF process, the ratio of the non-adhesive region was calculated as follows:

(1) $$RONAR=\frac{A_{NAR}}{A_{total}},$$

where $A_{NAR}$ is the area of the non-adhesive region, and $A_{total}$ is the total area photographed by the optical microscope. In this study, the average for each section of the RONAR was calculated. Furthermore, the RONARs of the FML specimen manufactured by the proposed MCF process were compared with those of the FML specimen manufactured by the commercial microwave oven with the PTFE mold in the previous study.

## 3. Results and Discussion

### 3.1. Stress–Strain Curves

**Figure 19:**
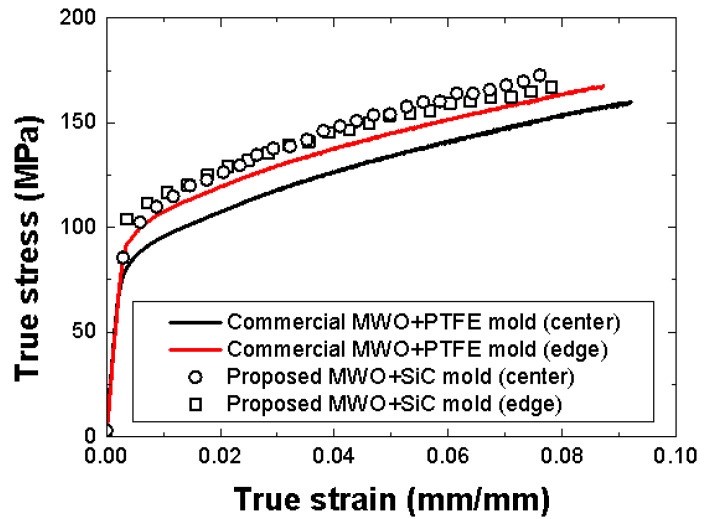
Stress–strain curves of FML specimens manufactured by the commercial [16] and proposed microwave ovens (MWOs).

Figure 19 shows the stress–strain curves of the FML specimens manufactured by the proposed MCF process and the MCF process proposed in the previous study. The stress–strain curves for the center and edge are those of the FML specimens made by cutting Position 3 and Positions 1 or 5, respectively, in Figure 14. When the commercial microwave oven and PTFE mold were used, the stress values of the FML specimen corresponding to the center and edge positions differed significantly. This implies that the stress at the center was higher than that at the edge because the center had a relatively high thermal expansion pressure when the PTFE mold pressurized the FML sheet. Furthermore, it was observed that the stress of the FML specimen manufactured in the previous study was lower than that of the FML specimen fabricated by the MCF process proposed in this study. This was because the commercial microwave oven could not precisely control the temperature of the FML sheet. It was verified that the stress of the FML specimen manufactured by the MCF process proposed in this study was higher than that of the FML specimen fabricated with the commercial microwave oven with the PTFE mold. In addition, the difference in stress between the specimen cut at the center and that cut at the edge was relatively marginal. This was because the proposed microwave oven used air pressure in the chamber rather than the thermal expansion pressure of the PTFE mold.

### 3.2. Comparison of Tensile Properties

**Figure 20:**
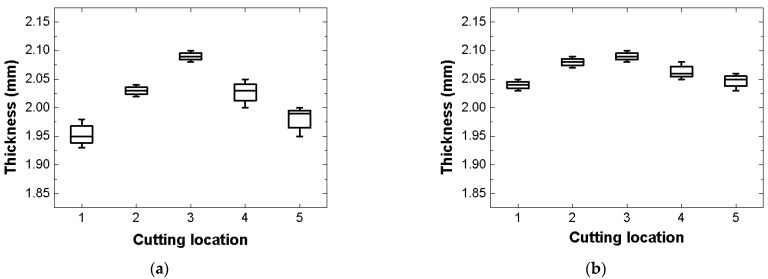
Thickness distributions of FML specimens according to cutting location: (**a**) Commercial microwave oven with PTFE mold; (**b**) Proposed microwave oven with SiC mold.

Table 2 shows the tensile properties of the FML specimens manufactured using the proposed microwave oven with the SiC mold and the commercial microwave oven with the PTFE mold. The tensile modulus, yield strength, and ultimate strength of the FML specimen manufactured using the proposed MCF process were approximately 0.18%, 0.28%, and 2.75%, respectively, which were higher than those of the FML specimen manufactured using the commercial microwave oven with the PTFE mold in the previous study. In addition, it was verified that the proposed MCF process in this study exhibited a relatively low standard deviation compared with the commercial microwave oven with PTFE mold. The tensile properties of the FML specimens manufactured using the proposed MCF process did not improve significantly. However, these would improve remarkably if the internal pressure of the proposed microwave oven is increased further considering the viscosity of the PP adhesive film. Figure 20 shows the thickness distributions of the FML specimens manufactured using the proposed MCF process and the commercial microwave oven with the PTFE mold.

The thickness of the FML specimen was determined by measuring the thickness of the five specimens according to each cutting location. When the commercial microwave oven with the PTFE mold was used, a relatively large difference in thickness according to the cutting location was observed. This was owing to the non-uniform thermal expansion pressure of the PTFE mold. However, it was verified that the thickness differences according to the cutting location could be decreased when the microwave oven with the SiC mold was used. Nevertheless, there was a marginal difference in the thickness of the FML specimen depending on the cutting location. This was because the melted PP adhesive film at the edge of the FML sheet could directly escape to the outside, whereas that at the center of the FML sheet could not do so because of its viscosity.

As a result, the FML specimen manufactured by the proposed MCF process showed a relatively uniform thickness compared with that fabricated by the commercial microwave oven with the PTFE mold. This resulted in relatively uniform tensile properties across the cutting locations.

### 3.3. Comparison of the RONARs

In this study, the non-adhesive areas of the FML sheets produced by the proposed MCF process and the MCF process using the commercial microwave with PTFE mold were quantified by the average RONAR according to FML section (see Table 3) and compared. For both the processes, the RONAR of FML section 2 was relatively lower than those of FML sections 1 and 3. This is considered to have occurred because the air inside the chamber made contact with the edge of the FML sheet to result in a relatively higher cooling rate than that for FML section 2 during the MCF process. As a result, the PP adhesive film did not have sufficient time to melt. Furthermore, owing to the characteristics of the microwave heating mechanism, a relatively large amount of heat was generated inside the FML sheet. This resulted in relatively sufficient melting of the PP adhesive film. A comparison reveals that the RONAR obtained with the proposed process is lower than that obtained with the MCF process using the commercial microwave oven with PTFE mold. For FML sections 1, 2, and 3, the proposed MCF process exhibited RONARs lower than those of the commercial microwave oven with PTFE mold by approximately 1.5758%, 0.0277%, and 3.8115%, respectively. In the case of FML section 2, it was verified that both the processes showed similar RONARs. Furthermore, in the case of FML sections 1 and 3, the proposed MCF process exhibited lower RONARs. This was because the temperature of the SiC mold of the proposed MCF process could increase to a value at which the PP adhesive film could be melted sufficiently compared with the PTFE mold.

Thus, it was verified that the proposed MCF process could improve the adhesion quality of the FML sheet more than the commercial microwave oven with PTFE mold. Furthermore, it was observed that the temperature difference between the edge and center of the FML sheet could be reduced using the SiC mold. It is considered that the MCF process proposed in this study can produce FML sheets of improved quality if the internal pressure of the proposed microwave oven is increased further and the temperature control cycle is optimized.

## 4. Conclusions

In this study, a microwave oven and SiC mold for manufacturing FML sheets were proposed to overcome the disadvantages of using a the commercial microwave oven with PTFE mold. The results obtained by the preliminary test of the mold, uniaxial tensile test, and microscope examination yielded the following conclusions:

(1). In the preliminary test of the mold, spark was not generated at the SiC mold during the MCF process, and the temperature of the FML sheet could rapidly attain the melting temperature of the PP adhesive film. It was also verified that the temperature could be maintained close to the set temperature by the temperature control system.
(2). The tensile modulus, yield strength, and ultimate strength of the FML sheet manufactured by the proposed MCF process were improved by approximately 0.18%, 0.28%, and 2.75%, respectively, compared with those of the FML sheet fabricated by the commercial microwave oven with PTFE mold. Notwithstanding the moderate numerical improvement, it is considered that the proposed MCF process can apply relatively uniform pressure. This is based on a comparison of the standard deviation of each tensile property and the thickness of the FML specimens according to cutting location.
(3). The RONARs of Sections 1, 2 and 3 of the FML specimens manufactured by the proposed MCF process were observed to be approximately 1.5758%, 0.0277%, and 3.8115%, respectively, lower than those of the specimens fabricated by the commercial microwave oven with PTFE mold. It was observed that the adhesion quality of the FML specimen manufactured by the proposed MCF process was higher than that fabricated by the MCF process using the commercial microwave oven with PTFE mold.

Although a simple flat-shaped SiC mold was used in this study, we plan to fabricate actual composite products with the proposed MCF process to review their structural performances and thereby, evaluate the applicability of the proposed MCF process in the industry.

## Figures and Tables

**Table 1:** Input power equation for controlling temperature.

Input Power (W)	Temperature Range (°C)
1100	$T<T_{set}-15$
$-\frac{220}{3}\left(T-T_{set}\right)$	$T_{set}-15\leq T\leq T_{set}$
0	$T>T_{set}$

*T*: measured temperature; $T_{set}$: set temperature.

**Table 2:** Tensile properties of FML specimens.

Process	Tensile Modulus (GPa)	Yield Strength (MPa)	Ultimate Strength (MPa)
Commercial MWO + PTFE mold [16]	32.2442 ± 0.7030	104.1680 ± 6.3918	161.7328 ± 6.306
Proposed MWO + SiC mold	32.3048 ± 0.7865	104.4612 ± 2.7765	166.3100 ± 5.7765

**Table 3:** RONARs of FML sheets manufactured by the proposed process and commercial microwave oven with PTFE mold.

Process	RONAR (%)		
	Section 1	Section 2	Section 3
Commercial MWO + PTFE mold [16]	6.7923	2.1442	10.0271
Proposed MWO + SiC mold	5.2165	2.1165	6.2156
